# Ensembles of Atoms, Ensembles of Species: Comparative Statistical Mechanics

**DOI:** 10.3390/e22060610

**Published:** 2020-05-30

**Authors:** Michael G. Bowler

**Affiliations:** Department of Physics, University of Oxford, Keble Road, Oxford OX1 3RH, UK; michael.bowler@physics.ox.ac.uk

**Keywords:** statistical mechanics, stochastic processes, alien species distributions

## Abstract

The methods of statistical physics are exemplified in the classical perfect gas—each atom is a single dynamical entity. Such methods can be applied in ecology to the distribution of cosmopolitan species over many sites. The analogue of an atom is a class of species distinguished by the number of sites at which it occurs, hardly a material entity; yet, the methods of statistical physics nonetheless seem applicable. This paper compares the application of statistical mechanics to the distribution of atoms and to the vastly different problem of distribution of cosmopolitan species. A number of different approaches show that these distributed entities must be in some sense equivalent; the dynamics must be controlled by interaction between species and the global environment rather than between species and many uncorrelated local environments.

## 1. Introduction

**Figure 1:**
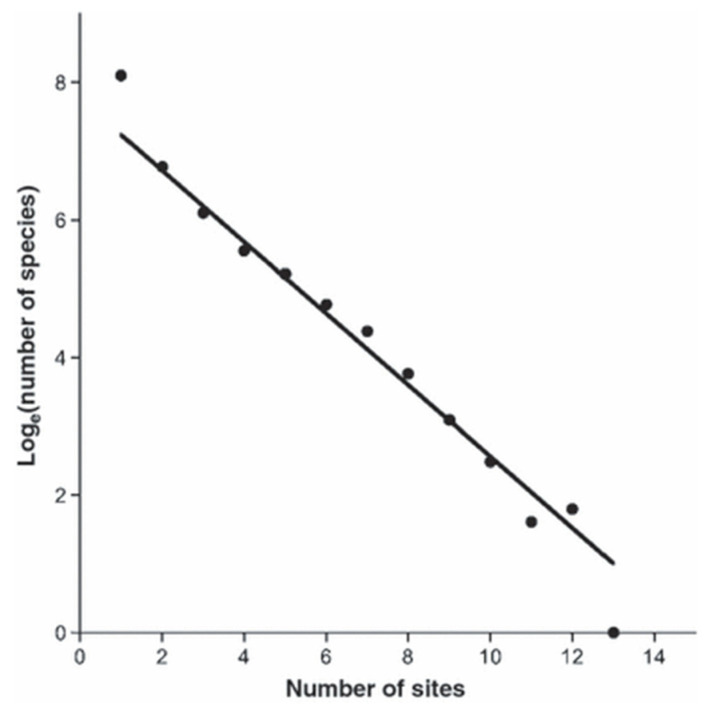
Illustration of the exponential distribution of the number of naturalized species as a function of the number of sites at which they are found. One species is found at 13 sites. This figure is taken from [1].

The analysis of a collection of data concerning the distribution of alien (naturalized) plant species over 16 sites worldwide revealed some marked and surprising properties [1]. Classifying species according to the number of sites at which they are found revealed, first, that the number of species found at *n* sites is distributed exponentially (*n* > 1); with over 5000 species, the mean of the exponential was ~1.9 occupied sites per species. One was found at 13 out of 16 sites, Figure 1.

Secondly, there exists no correlation between physical separation and the number of occupied sites. Thirdly, the number of pairs of sites sharing a species is exponential in the number shared; similarly for triplets of sites. The details are to be found in [1,2]; for this paper, it suffices to concentrate on the exponential distribution of the number of species as a function of the number of sites at which each is found. To a statistical mechanic, such an exponential suggests a Boltzmann distribution; a sufficiently well-informed ecologist might think of MacArthur’s broken stick model (for species abundance) [3] and an aficionado might think of maximum entropy. None of these approaches directly addresses the underlying biological machinery.

It seems surprising that a process as complicated as naturalization over the globe of these alien species admits such a simple description. To some, it may be even more surprising that the distribution is exponential rather than the log series familiar from species abundance distributions. It seems that the interactions of species with the local environments at which they are found must be correlated on a global scale. In this paper, various approaches to the statistical mechanics of the abundance of alien species are compared with the statistical mechanics of the ideal perfect gas, in the hope that, if not actually enlightening, the comparison may at least prove suggestive.

## 2. Microcanonical Ensembles

### 2.1. The Classical Perfect Gas

The statistical treatment of the classical perfect gas as a microcanonical ensemble is familiar and repeated here solely for comparison with a putative microcanonical ensemble of species. *N* identical atoms are confined and energy levels $E_{i}$ are available to all equally. Both the total energy and the number of atoms are conserved. From here on it is a matter of combinatorics. Assigned to each energy level are a number $n_{i}$ of atoms and the probability of any particular arrangement {$n_{i}$} is obtained from the number of different yet equivalent ways of arranging such a set. The number of different ways of ordering the atoms in {$n_{i}$} is N!, but the atoms that are members of any single $n_{i}$ can be arranged in $n_{i}!$ different ways, all of which are equivalent for the underlying physics and should be counted only once. Thus, the number of ways of making physically relevant arrangements is

(1) $$W\left(\left\{n_{i}\right\}\right)=\frac{N!}{\Pi n_{i}!}$$

where Π denotes the continuous product. The maximum of this weight, taken over all $n_{i}$, gives the most probable configuration, subject to the constraints that the total number of atoms *N* is fixed and that the total energy $\underset{i}{\sum }n_{i}E_{i}$ is also fixed. The usual procedure is to maximise the (natural) logarithm of the weight and to approximate ln(*n*!)≅*n*ln*n*-*n*. The constraints are imposed by the method of undetermined multipliers (a good discussion is to be found in [4]) and so the function to be maximized is

(2) $$N\ln N\;-\underset{i}{\sum }\{n_{i}\ln n_{i}-\lambda n_{i}-\mu n_{i}E_{i}\}$$

Differentiating with respect to $n_{i}$ and equating to zero, the most probable values are

(3) $$n_{i}=e^{\lambda }e^{\mu E_{i}}$$

The undetermined multipliers λ and μ are determined, a posteriori, by the number of atoms and total energy.

Not only are all the atoms equivalent, but any given {$n_{i}$} has to be reached somehow. In applying this treatment to an (idealized) gas, there are implicit dynamical notions. Atoms scatter with the conservation of energy and every scattering changes the configuration $\left\{n_{i}\right\}$. The microcanonical ensemble explores configurations of which the vast majority correspond to (3).

### 2.2. Alien Species

The ecological problem of how alien species are distributed over many sites can be addressed in the same way [1]. Each species is classified by the number of sites at which it is found and $s_{n}$ is the number of species found at *n* sites. Thus, *n* is, in some sense, equivalent to the $E_{i}$ and $s_{n}$ to the $n_{i}$ in the physics of the classical perfect gas. The total number of (alien) species to be described is fixed and the total number of alien colonies $\underset{n}{\sum }ns_{n}$ is also fixed. This is the conserved quantity called the *alien footprint* in [1,2]. The combinatorial weight is then constructed in the same manner as for the gas and maximized in the same way, subject to the constraints. The result is

(4) $$s_{n}=e^{\lambda }e^{\mu n}$$

It should be noted that in the absence of constraints, the values of $s_{n}$ would be all the same; in setting up the combinatorial weight each species is treated as equivalent to every other, in the same way that atoms are treated as equivalent in a gas. These assumptions were made to yield the observed exponential [1].

Each atom belongs to an energy level; each species belongs to some *n* sites. The probability of finding $s_{n}$ is the ratio $p_{n}=s_{n}$/S; thus, the exponential distribution is also information Maximum Entropy. This might appear to be an elegant and simple solution to the ecological problem addressed, but it is not sufficient. The equivalence of the species was essential in setting up the combinatorial weight, and in the language of Maximum Entropy, this equivalence corresponds to a uniform prior. The configuration characterized by (4) has to be reached somehow; it is not just a matter of MaxEnt. It is important to examine the dynamics, established or hypothetical. This same equivalence is characteristic of dynamics that yield exponential distributions.

## 3. Dynamics

### 3.1. Atomic Collisions

In this approach, the dynamics implicit in the microcanonical treatment are explicit and at the heart of the argument. The Boltzmann distribution emerges, and yet the treatment bears no resemblance to the microcanonical ensemble and no resemblance to standard MaxEnt arguments.

The atoms of an ideal gas confined within some container scatter and scatter again among themselves, subject to the conservation laws of Newtonian mechanics. For the present purposes, the most important of these is conservation of energy, valid not only in some average sense, but absolutely. Raising a given atom from one energy level to another conserves the number of atoms but does not conserve energy. However, in a scattering process, two atoms shift energy levels and this can and must conserve energy. This simple consideration alone is sufficient to enforce, in equilibrium, the Boltzmann distribution. An atom in level i scatters with an (identical) atom in level j, the atoms ending up in levels k and l. In equilibrium, the rates for this reaction and its inverse must be the same, and this imposes the condition

(5) $$n_{i}n_{j}=n_{k}n_{l},\;\mathrm{or}\;\ln n_{i}+\ln n_{j}=\ln n_{k}+\ln n_{l}$$

subject to the constraint

$$E_{i}+E_{j}=E_{k}+E_{l}$$

The solution to these equations is

(6) $$\;n_{i}=\alpha e^{\beta E_{i}}$$

where the constants α and β are determined by normalisation and the temperature of the gas. In Section 3.3 below, I speculate on applying this treatment in reverse to the statistical mechanics of alien species. (I came across this treatment, which seems little known, over 40 years ago, and have no recollection of where I found it. It is discussed in more detail in [5]).

### 3.2. Species Master Equations

A species can be characterized by an integer. In considerations of species abundance distributions, this integer is the number of individuals in a given region or a given area. The application of statistical mechanics and MaxEnt to the problem of species abundance distributions has a long history [6], but here we are concerned, not with species abundance, but with an abundance of species. The alien species are characterized by the number of sites to which they are alien, yet present. A species found at *n* sites is characterized by that number, even though some sites are separated by intercontinental distances; the number of such species is denoted by $s_{n}.$ There is a dynamical aspect, because the individual species alien to the sites have migrated there by one means or another. A simple possible description of the dynamics supposes that individual sites open and close to individual species; if a species at *n* sites loses a single site, then $s_{n}$ decreases by 1 and $s_{n-1}$ increases by 1. One can consider a master equation for the hypothesis that the only dynamical processes are the gaining or losing of a site by a single species [2].

(7) $$\frac{ds_{n}}{dt}=-s_{n}\left(r_{n}^{+}+r_{n}^{-}\right)+r_{n-1}^{+}s_{n-1}+r_{n+1}^{-}s_{n+1}$$

where $r^{+}$ and $r^{-}$ are the rates at which a species gains or loses a site. (Just such equations have been employed in describing species abundance distributions [7].) In an equilibrium, the following relationship must hold:

(8) $$s_{n}=\frac{r_{n-1}^{+}}{r_{n}^{-}}s_{n-1}$$

and (because *n* is integer) can be iterated. It is immediately obvious that if the ratio of rates in this equation is independent of the number of sites *n*, then the number of species at *n* sites is exponential in *n*. The simplest realization of this is to have both rates individually independent of *n*. This agrees with the condition imposed in the combinatorial approach: each species, regardless of its number of sites, is equivalent to every other. Then

(9) $$s_{n}=s_{0}R^{n}$$

where *R* is the ratio $r^{+}/r^{-}$ and is independent of *n.*

If these dynamics (or an approximation thereto) are the explanation for the exponential distribution of alien species over sites, then the following conditions must be satisfied. The rates at which an individual species is added to or eliminated from any site do not depend significantly on the species or on the site, and in particular, do not depend on the number of sites at which that species is present. This presents a marked contrast to the use of the master equation in species abundance distributions, where it is supposed that the rates at which individual species gain or lose individuals (individual trees usually) that are linearly dependent on the number (of trees) of a given species present in the plot. In neither case are there correlations. In the dynamics of forest populations, the conditions of (9) might be realized by an inebriated forester.

It seems natural enough for the rate at which a species colonizes a new site to be independent of the number at which it already exists; it seems less acceptable for the rate at which a species is lost from a site to be independent. If the physics within the master equation is a reasonable approximation, there must be fluctuations in the environment (in the most general sense) occurring at a (reasonably) steady rate, but each fluctuation affecting specifically a single species and a single site. This might be an example of the notion of ‘idiosyncracy’ formulated in [8]; see also [1].

### 3.3. Species Collisions?

The exponential distribution in *n* of $s_{n}$ results from the microcanonical treatment in the same way as the Boltzmann distribution for a gas. The Boltzmann distribution also results from simple mechanics applied to the picture of scattering atoms. For species, if $s_{n}$ is distributed exponentially with *n*, then automatically, the relation

$$s_{i}s_{j}=s_{k}s_{l}$$

holds, provided that

i+j=k+l

Is it possible that these relationships reflect a dynamic totally different from that of the master equation and if so what would be the nature of the underlying biology? This possibility is easily explored and any machinery would certainly involve correlations; quite different from uncorrelated events in the master equation for species abundance. For a gas, the underlying process is the scattering of a single atom in state *i* having energy $E_{i}$ from a single atom in state *j* having energy $E_{j}$, with conservation of energy. The rate at which this occurs depends on the product of the number of atoms in state *i* and the number in state *j*. The removal of an atom in state *i* reduces $n_{i}$ by one unit, carrying with it a single unit of the energy $E_{i}$. If there is an analogous process in the statistical mechanics of alien species, then the number of species $s_{i}$ at *i* sites is the analogue of $n_{i}$. Removal of a single species reduces $s_{i}$ by one unit, carrying with it the number of sites at which it is found, the number analogous to the energy of a single atom and the contribution of this species to the alien footprint. Thus, this analogy requires some interaction among pairs of species, however indirect, that conserves the alien footprint. The role attributed above to the products $s_{i}s_{j}$ is a further instance of the identity condition for the species characterized by their number of sites. There is also the requirement that removal of a species that contributes to $s_{n}$ occurs effectively at the same moment at all n sites at which it was found. Thus, a dynamic based on this admittedly speculative notion is geographically global. Convulsions in the environment (climate change is merely a possible example) kill off (or favor) an alien species everywhere, and this rate is independent of the number of sites. To complete the dynamic, species from two classes are so removed and replaced by two species in two different classes, subject to conservation of the alien footprint. More realistically, some global variation in the environment, in the most general sense, could be thought of as changing the number of sites at which the two species are found, subject to conservation of the number of sites. Fanciful though it may be, these entities of species naturalized at sites scattered globally can be treated in terms of a precise analogy with the collisions of atoms. The species ‘scatter’ from each other.

### 3.4. A Diversion into Population Dynamics

If species in some ecological guild are characterized by the number of individuals, then the familiar log series distribution results from the master Equation (7), by setting both $r^{+}$ and $r^{-}$ proportional to the population *n.* Then, the number of species with population *n* is of form

$$s_{n}=\frac{\alpha }{n}e^{-\beta n}$$

and consequently

$$\left(ns_{n}\right)\left(ms_{m}\right)=\left(ks_{k}\right)\left(ls_{l}\right)$$

provided that

n+m=k+l

This can be interpreted as expressing equilibrium where $ns_{n}$ elements ‘scatter’ from $ms_{m}$; the dynamical elements are the individual trees (or spots where trees are to be found). The contrast between this case and the failure of alien sites to act as independent dynamical elements or entities is quite clear, but we have another way of understanding the emergence of the log series distribution underlying species abundance distributions, additional to those discussed in [6].

### 3.5. Atoms—The Master Equation?

To complete the comparative statistical mechanics of atoms and alien species, it is natural to ask whether atoms can be treated with an analogue of the master equation for species (7). We can write down the analogous equation, but it is only applicable under very restrictive conditions. Consider the equation

(10) $$\frac{dn_{i}}{dt}=-n_{i}\left(r^{+}+r^{-}\right)+r^{+}n_{i-1}+r^{-}n_{i+1}$$

This is a transcription of the master Equation (7), that yields an exponential dependence of $s_{n}$ on *n*, with $n_{i}$ replacing $s_{n}$ and *i* replacing *n*. Both $n_{i}$ and *i* are integers, but here is the rub- *n* is a dynamical variable as well as being a label. The suffix *i* is merely a label and the dynamical variable is the energy labelled by *i*, $E_{i}$.

Nonetheless, since *i* is an integer, the equilibrium condition can be iterated to yield

(11) $$n_{i}=n_{0}{\left(\frac{r^{+}}{r^{-}}\right)}^{i}$$

This is a formal solution to the equation for equilibrium, but is of little value unless the integer *i* can be related to the corresponding energy $E_{i}$. The solution is exponential in *i*; if we have $E_{i}=i\varepsilon$, then $n_{i}$ is exponential in $E_{i}$. There are two unsatisfactory things about this proposition. First, the treatment is restricted to uniformly spaced energy levels and secondly, whatever the mechanism, atoms only shift between adjacent energy levels. It is too restricted and artificial, and the simple master Equation (10) had best be abandoned in this context.

## 4. Discussion

These comparisons of the statistical mechanics of gases on the one hand, and the postulated statistical mechanics of the abundance of species on the other, make it very clear that an understanding of the underlying machinery leading to a particular distribution has an importance that is often overlooked. The microcanonical ensembles do not involve the machinery underlying the dynamics of a gas, nor the dynamics of migrating species. The microcanonical ensemble is equivalent to maximum entropy with a uniform prior—why is the prior uniform? This must be looked for in the underlying dynamics [6]. For the gas, where the elements are individual atoms, the essential dynamics are well understood. For cosmopolitan species, it is clear that the dynamics remain mysterious. The comparison of these two cases is educational and has contributed one suggestive result. The detailed balance treatment of the gas (Section 3.1) can be transcribed in the symbols for migrating species and it does not appear that this could not be realized in the real world—a global process in which, if a species vanished from one alien site, it would (more or less simultaneously) vanish from many, as a result of some kind of global convulsion (Section 3.3). In the contrasting case, (micro-) convulsions are local; they would need to be directed to a specific species and that species at a specific site. Even so there are global aspects—if a given site is vulnerable all others are not (and of course the alien footprint is a global constraint). These explanations may be fanciful, but show how the exponential distribution of Figure 1 might be reached through global effects or targeted local effects. The exponential distribution exists and requires that the elementary entities, the species that are scattered across the globe, are in some sense equivalent and the number of sites does not matter at all. The origin of this intriguing ecological curiosity remains to be determined.
